# Vehicle–Road Wear Microplastics: Fragmented Understanding of Their Impacts on Environment

**DOI:** 10.34133/research.1323

**Published:** 2026-06-19

**Authors:** Yongsheng Hai, Xuan Yang, Siqi Luan, Zepeng Fan, Yulin He, Qilin Yang, Guoyang Lu, Chaohe Wang, Dawei Wang, Alberto Carpinteri

**Affiliations:** ^1^ School of Transportation Science and Engineering, Harbin Institute of Technology, Harbin 150090, PR China.; ^2^ Chongqing Research Institute of HIT, Chongqing 401135, China.; ^3^ Department of Architecture and Civil Engineering, City University of Hong Kong, Kowloon Tong, Hong Kong, China.; ^4^ Institute of Highway Engineering, RWTH Aachen University, Aachen 52074, Germany.; ^5^ Department of Structural, Geotechnical and Building Engineering, Politecnico di Torino, Corso Duca degli Abruzzi 24, 10129 Torino, Italy.

## Abstract

Vehicle–road wear microplastics (VRWMPs) are microplastic-sized polymer-containing particles generated from the vehicle–road system, including tire–road wear particles (TRWPs) as the dominant composite class and other polymer-bearing wear debris (e.g., road marking wear). Their complex chemical composition and wide particle size distribution pose potential ecological risks. However, their impacts on ecosystems have long been overlooked because overlapping terminology (e.g., TRWP versus broader non-exhaust emissions) and nonstandardized characterization methods hinder cross-study comparability, while a tire-centered research focus and limited field monitoring obscure the contribution of pavement materials and realistic exposure scenarios. Existing studies largely emphasize tire-derived contributions, while the role of pavement materials remains underrepresented, resulting in an incomplete understanding of VRWMP formation mechanisms. In addition, limited long-term and systematic monitoring data constrain current knowledge of VRWMP migration, transformation, and environmental risks. From a road engineering perspective, this review synthesizes the full lifecycle of VRWMP, from generation to environmental fate. It focuses on formation mechanisms, preparation and characterization methods, migration, and transformation processes within roadway systems, and associated ecological and human health effects. Evidence indicates that VRWMP generation is jointly controlled by tire characteristics and pavement materials, yet a standardized characterization framework is still lacking. The migration and transformation of VRWMP are difficult to model due to data scarcity and pronounced regional variability related to geography, climate, and traffic conditions. Soil and aquatic environments represent major sinks, and exposure pathways such as inhalation may induce adverse biological effects, including oxidative stress and DNA damage. With the increasing complexity of pavement materials, establishing full-chain control of VRWMP, from generation to environmental fate, is becoming an urgent research priority. This review provides a scientific basis for advancing cleaner and more sustainable transportation infrastructure.

## Introduction

Microplastic pollution has been recognized as one of the most pressing environmental challenges of the 21st century [1]. Microplastics can reduce feeding efficiency [2], alter energy allocation [3], cause gastrointestinal injury and inflammatory responses [4], and induce oxidative stress that impairs growth and reproduction [5]. In addition, they act as carriers of polymer additives and environmental contaminants [6], extending potential impacts from physical particle effects to chemical exposure. Humans may be exposed to microplastics through dietary intake and inhalation [7], with dietary uptake having increased between 1990 and 2018 across 109 major developing and industrialized countries [8]. Among the diverse sources of environmental microplastics, those generated from the vehicle–road system, referred to herein as vehicle–road wear microplastics (VRWMPs), represent a uniquely persistent, continuously emitted, and compositionally complex category that has remained disproportionately underrecognized in environmental risk assessments. VRWMP encompass Tire–road wear particles (TRWPs) as their dominant composite class, along with other polymer-bearing wear debris from road markings and brake systems, as well as particles derived from pavement wear, clutch wear, solid waste fillers, winter de-icing and maintenance operations, and road dust. Unlike many consumer-derived microplastics that enter the environment through sporadic or point-source pathways, VRWMPs are generated ubiquitously across road networks during routine traffic operations, leading to widespread and chronic release. Their heterogeneous nature, combining tire rubber, mineral aggregates, binder residues, and additive-derived chemicals, gives rise to physicochemical properties and environmental behaviors that cannot be reliably inferred from studies of pristine tire particles or general microplastic mixtures alone.

With global plastic production continuing to rise, the accumulation of microplastics in the environment has become an urgent global concern. Research estimates that more than 170 trillion microplastic particles are floating in the oceans [9], while soil concentrations can reach up to 3.573 × 10^6^ particles per kilogram [10]. Annual microplastic emissions to the atmosphere are estimated at 9.6 ± 3.6 Tg year^−1^ [11]. Consequently, microplastic pollution is widely recognized as one of the most pressing environmental challenges of the 21st century [12,13], with related research rapidly emerging as a major frontier in environmental science. The urgency of this issue has drawn the highest-level international attention: The United Nations Environment Assembly has adopted a landmark resolution “Eliminating Plastic Pollution: Towards a Legally Binding Instrument” to advance coordinated global governance [14].

### Vehicle–road wear microplastics

Among various sources of microplastics, TRWP has attracted considerable attention due to its substantial emissions. TRWP is a heterogeneous mixture produced by the mechanical abrasion of tire treads as they interact with road surfaces [15]. It comprises not only rubber from the tire itself but also embedded pavement materials, mineral particles, and other traffic-related wear debris, including those from brakes and clutches [16,17]. Notably, TRWP represents only one major category within the broader concept of VRWMP, which also encompasses particles originating from brake wear, clutch wear, and other road traffic-related abrasion processes. Therefore, beyond TRWP, a comprehensive understanding of vehicle–road wear MPs is essential, and the following sections provide an overview of their sources, characteristics, environmental occurrence, and potential impacts.

Quantitative assessments underscore the substantial contribution of VRWMP to environmental microplastic pollution: Approximately 84% of atmospheric microplastics are attributed to road-related emissions [18]. Globally, annual VRWMP generation is estimated at 1.327 million tons in the European Union, 1.12 million tons in the United States, and 0.756 million tons in China, and per capita generation ranges from 0.2 to 5.5 kg [19]. As reported in the literature, VRWMP can contribute up to 53.9% of the microplastics in the environment [20]. Collectively, these authoritative findings highlight that VRWMP is a major source of environmental microplastics, and its potential risks warrant urgent attention and prioritization in environmental governance.

**Fig. 1:**
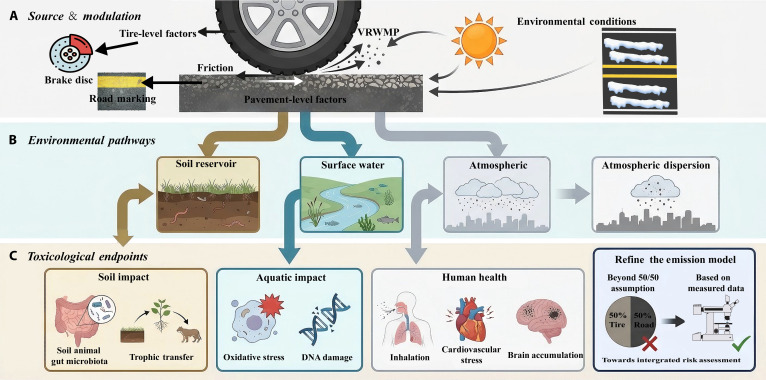
From generation to risk: Pathways and toxicological impacts of VRWMP: (A) Generation mechanism of VRWMP. (B) Impact of VRWMP on the ecological environment. (C) Human health impacts of VRWMP.

Considering the critical severity of VRWMP pollution and its intrinsic association with road infrastructure, this article presents a critical review of its lifecycle environmental behavior from the distinct perspective of road engineering. We aim to establish a systematic framework spanning from “source generation” to “terminal risk”, specifically dissecting how road characteristics govern the physicochemical properties of VRWMP at the source (Fig. 1A). Furthermore, this review synthesizes characterization methodologies tailored to the complex heterogeneity of VRWMP and scrutinizes its migration and transformation pathways across atmospheric, aquatic, and terrestrial matrices (Fig. 1B), alongside the cumulative risks posed to roadside ecosystems and human health (Fig. 1C). This systematic synthesis seeks to deepen the industry’s understanding of the VRWMP environmental footprint, providing a scientific foundation for material optimization in green, low-carbon pavement design and informing holistic environmental management strategies.

### Literature selection and review strategy

A 2-tier search strategy was implemented using the Web of Science Core Collection (2000–2025), limited to peer-reviewed articles and reviews in English. The scope included studies addressing the generation, characterization, occurrence, transport, fate, toxicity, and mitigation of tire road wear particles, while excluding works focused exclusively on exhaust emissions or unrelated dust sources.

The core dataset was constructed using terminology specific to tire and road wear particles (e.g., TRWP, tire/tyre wear particles, tire-derived microplastics, and related expressions), combined with environmental and toxicological descriptors such as microplastics, ecosystems, ecotoxicity, occurrence, transport, fate, monitoring, leachates, and key compounds including 6PPD and 6PPD-quinone. This targeted approach yielded 906 records and served as the primary corpus for bibliometric analysis.

To address potential limitations of narrow terminology, a supplementary dataset was developed by incorporating broader descriptors commonly used in adjacent disciplines such as road engineering, atmospheric science, and urban hydrology. These included terms related to road dust, non-exhaust emissions, roadway particles, runoff contaminants, pavement and asphalt wear, and rubberized asphalt leachates, again combined with the same environmental and toxicological keywords. This expanded search retrieved 4,432 records. The supplementary dataset was not used for bibliometric mapping but was employed to assess the extent of terminological variation across fields.

**Fig. 2.**
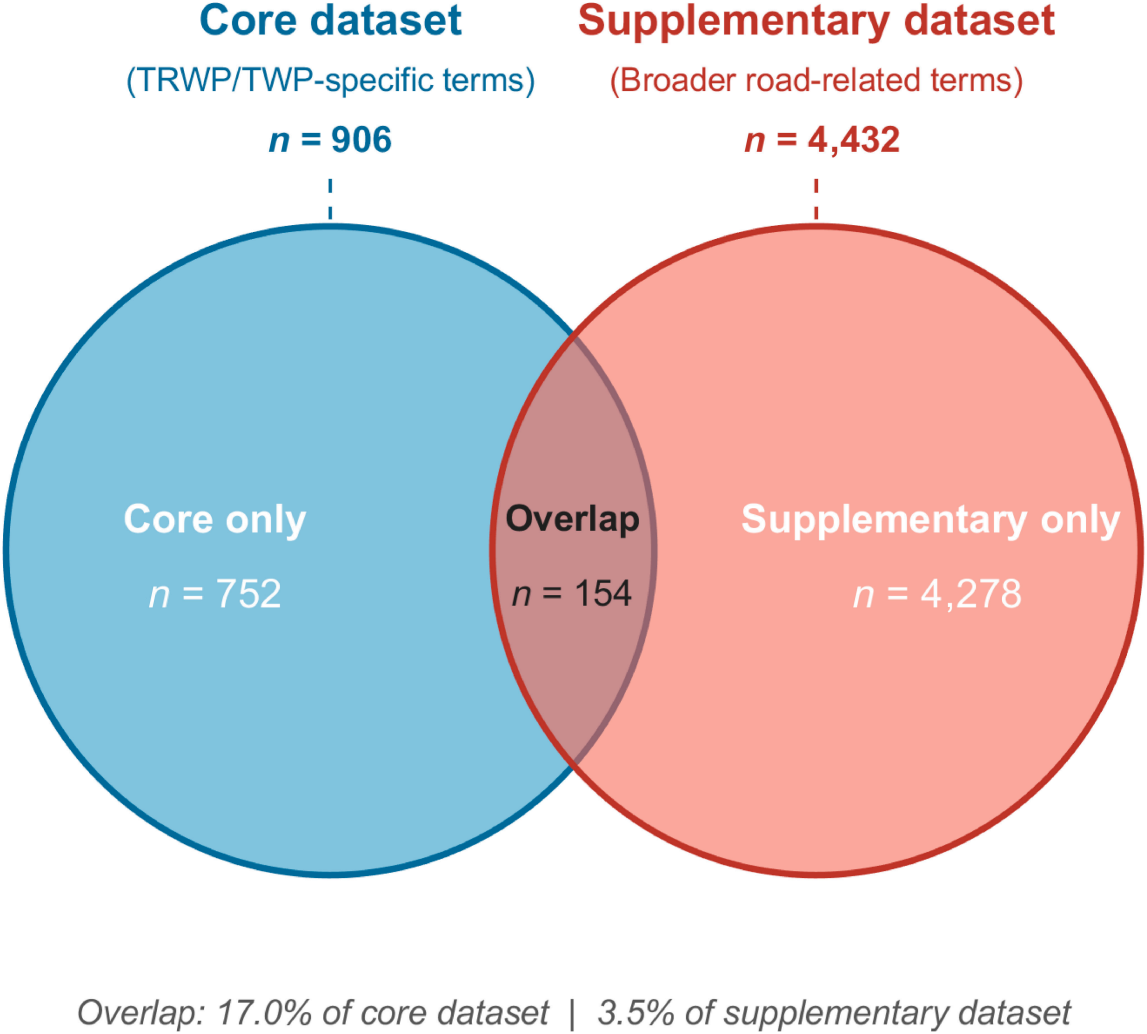
Venn diagram: Keyword co-occurrence map of the core dataset was performed using CiteSpace (v6.4.R2). The keyword co-occurrence network (455 nodes, 1,104 links; density = 0.0107) is centered on terms such as tire wear particles, microplastics, toxicity, environment, fate, sediment, runoff, and oxidative stress, indicating that the research domain is primarily structured around environmental occurrence, pollutant transport, ecotoxicity, and emerging chemical risks (Fig. 3).

**Fig. 3.**
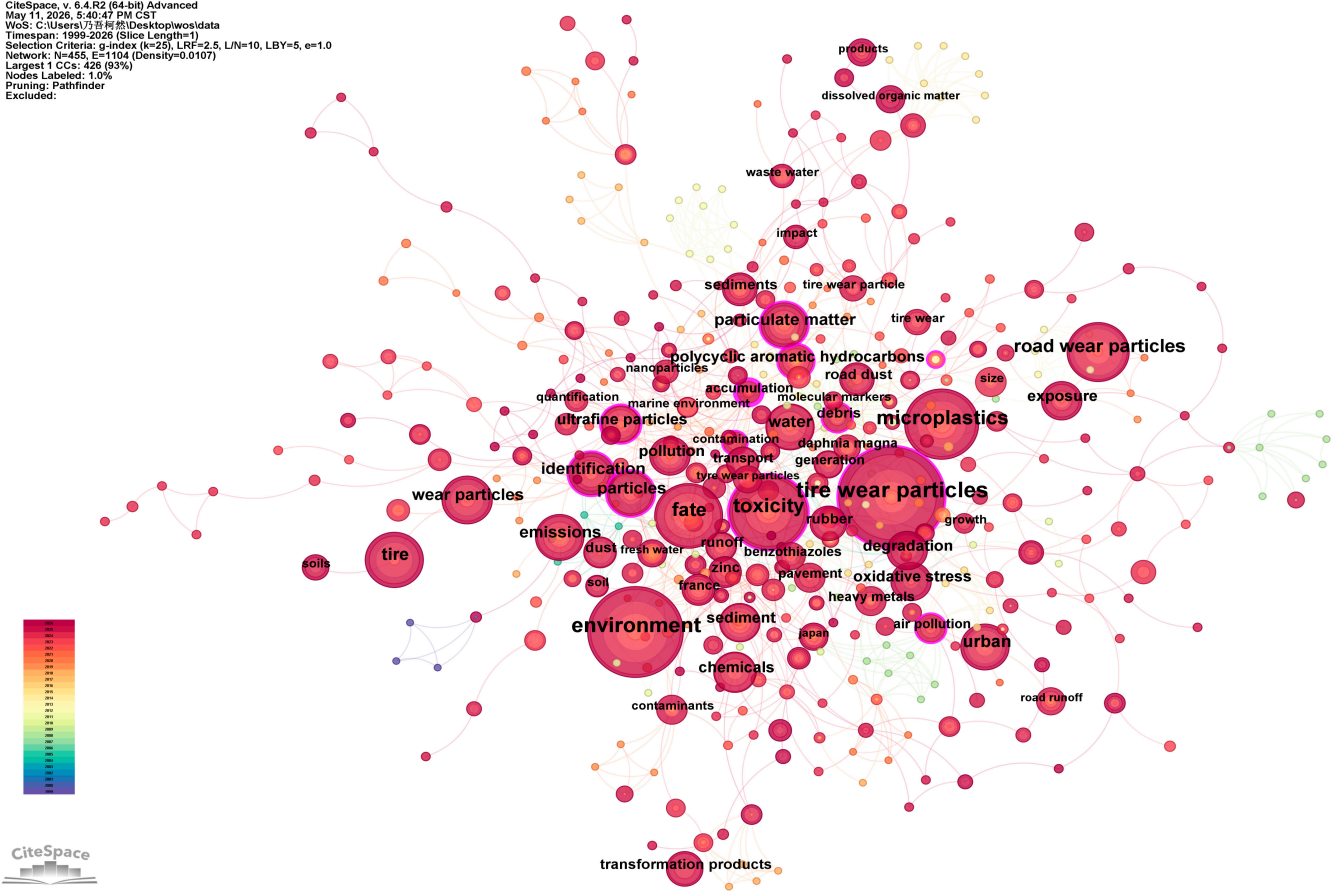
Keyword co-occurrence map: Keyword co-occurrence network map of VRWMP-related literature retrieved from the Web of Science Core Collection. Each node represents a keyword, with node size proportional to its occurrence frequency, and connecting lines indicate co-occurrence relationships between keywords.

Comparison of the 2 datasets using a Venn diagram (Fig. 2) revealed limited overlap: Only 154 records were shared, corresponding to 17.0% of the core dataset and 3.5% of the supplementary dataset. In contrast, 752 records were unique to the core dataset, while 4,278 records were retrieved exclusively through broader terminology. This pronounced asymmetry demonstrates substantial fragmentation in terminology across disciplines. It indicates that a large body of relevant research, particularly from road engineering and non-exhaust emission studies, remains weakly connected to the TRWP/VRWMP-focused literature. This helps explain the discrepancies between thematic coverage and keyword-based bibliometric patterns (Fig. 3).

**Fig. 4.**
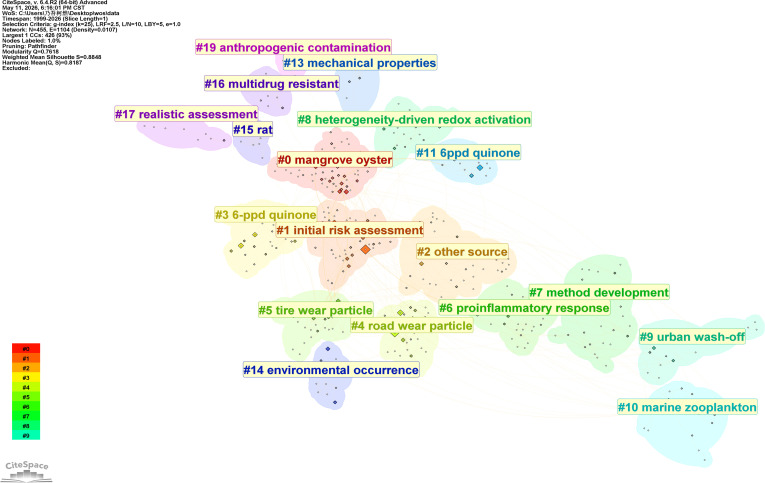
Keyword clustering diagram: Visualization of keyword co-occurrence clusters derived from bibliometric analysis of VRWMP-related literature. Different colors represent distinct thematic clusters, with larger node sizes indicating higher keyword frequency.

Cluster analysis further confirms this structure (Fig. 4). Major clusters include tire wear particle (TWP), road wear particle, environmental occurrence, 6PPD-quinone, urban wash-off, marine zooplankton, proinflammatory response, and method development. These clusters emphasize environmental distribution, exposure pathways, biological effects, and analytical approaches. In contrast, topics related to pavement materials, tire–road contact mechanics, abrasion mechanisms, and design-oriented mitigation are weakly represented, with “mechanical properties” appearing only as a minor peripheral cluster.

Combined with the Venn analysis, these results indicate that the VRWMP field is not lacking in research activity but is structurally fragmented. Environmental and toxicological studies dominate the TRWP/TWP-defined literature, whereas road-material and generation-mechanism studies are largely developed under separate terminological systems, resulting in limited cross-disciplinary integration. Given the hierarchical relationship between TRWP and VRWMP, and the structural knowledge gap identified above, the term VRWMP is adopted hereafter as the unified terminology throughout this review to ensure conceptual consistency. Accordingly, all subsequent references in this review will consistently employ the term VRWMP unless otherwise specified.

Overall, the bibliometric evidence reveals a pronounced knowledge gap: Despite the recognized contribution of VRWMP to non-exhaust emissions, research from road engineering and materials perspectives remains insufficient, such as diffusion and migration mechanisms, decomposition and ecotoxicity, influencing factors (including climate, pavement type, and traffic conditions), and tracking. Although these scientific questions are of vital importance in environmental studies, exploring VRWMP and its derivative problems in pavement will help enhance the research scientifically of the study. Focusing on the pavement system not only facilitates source identification and attribution of VRWMP but also supports a closed-loop research framework linking particle generation, migration, environmental fate, and mitigation strategies. This gap underscores the necessity of positioning VRWMP research within a road-material framework, with particular emphasis on abrasion behavior, material composition, and design-oriented mitigation strategies. Accordingly, this review aims to synthesize current knowledge, identify research trends and gaps, and explore implications for cleaner material design and mitigation approaches within a unified vehicle–road interaction framework.

## Mechanisms of VRWMP Generation

**Fig. 5.**
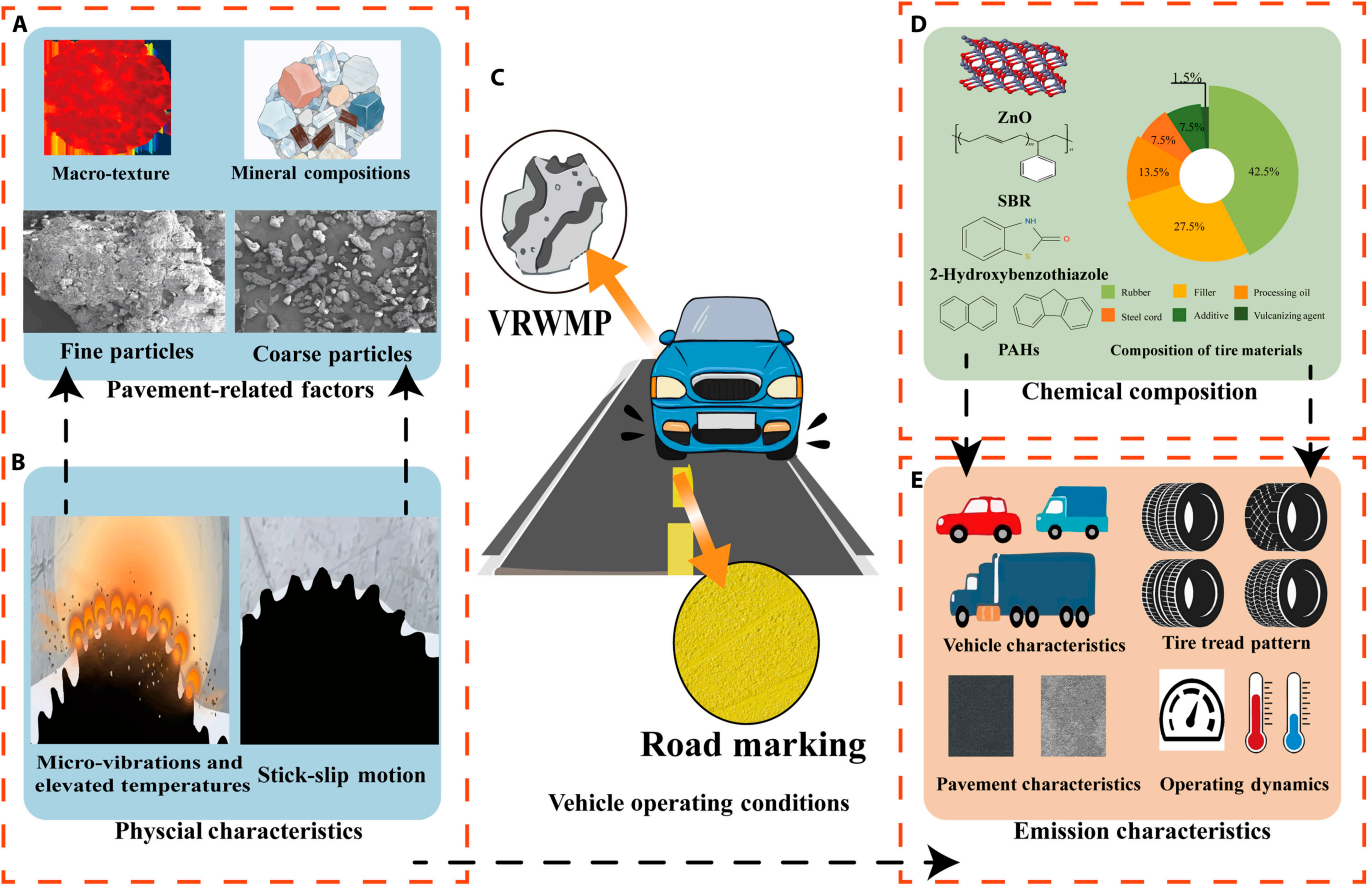
Schematic of VRWMP generation mechanisms. (A) Pavement-related factors, covering material composition, surface macro/microtexture, and particle size. (B) Physical characteristics: The VRWMP formation process involves coupled mechanical and thermal interactions between the tire and the pavement, which generate heterogeneous aggregates ranging from nanometers to hundreds of micrometers. (C) Vehicle operating conditions. (D) Chemical composition of VRWMP, encompassing tire- and pavement-derived constituents. (E) Emission characteristics: Tire-related factors, including tread material composition, tire type, and service conditions such as pavement type and operating dynamics.

VRWMPs are generated through the rolling shear interaction between tires and road surfaces. Relative to the pristine tire tread, the heat and friction induced by tire–road interaction can alter the chemical signature of the resultant particles. Moreover, VRWMP generated during tire–road contact can also contain admixtures of media and pavement materials, making VRWMP in real road environments primarily a heterogeneous aggregates Comprising tire rubber and mineral particles [16,19]. Consequently, the formation mechanism of VRWMP is governed by factors operating at both the tire level and the pavement level. Tire-level factors include tread material characteristics (material composition, service life, etc.), vehicle type (passenger cars, trucks, motorcycles, etc.), and driving behaviors (load, acceleration, braking, turning, skidding, etc.). On the pavement side, road surface characteristics (material composition, texture, etc.) and the influence of pavement media (road dust, sediment, standing water, snow accumulation, ice, etc.) play significant roles. This section comprehensively reviews recent research on the formation mechanisms of VRWMP, focusing on its physical and chemical properties, as well as emission characteristics, and explores the key factors influencing its formation (Fig. 5).

### Physical characteristics

The physical characteristics of VRWMP include morphology, particle size, and density, which significantly influence their environmental fate. The size distribution of VRWMP ranges from nanometers to hundreds of micrometers, with coarse particles predominating and tending to deposit on road surfaces or enter aquatic environments via stormwater runoff. It is estimated that only 0.1% to 10% of VRWMP particles smaller than PM10 become airborne [21]. The size distribution and concentration of VRWMP are influenced by friction-adhesion mechanisms, where the tire surface energy and wear patterns play crucial roles. Studies indicate that coarse particles larger than 4 μm arise from stick-slip motion between tires and road surfaces, whereas fine particles are formed due to the evaporation and rapid condensation of volatiles on tire surfaces, driven by micro-vibrations and elevated temperatures [22]. Table 1 summarizes the particle size distributions and testing methods for VRWMP generated in real road environments and road simulators. It is evident that VRWMP produced by existing road simulators still exhibit significant differences in particle size distribution compared to those in real road environments.

**Table 1.** VRWMP particle size distribution

VRWMP category	Formation of VRWMP	Driving conditions	Testing methods	Size range	Number of peaks	Peak value
Coarse particles	Real-world road environment [16]	-	Laser diffraction analyzer	4–280 μm	Unimoda	50 μm
		-	Transmission optical microscope	4–265 μm	Unimoda	50 μm
	Road simulator [16]	-	Laser particle size analyzer	5–220 μm	Unimoda	75 μm
		-	Transmission optical microscope	4–350 μm	Unimoda	100 μm
Fine particles	Tire simulator [140,141]	80 km/h	APS	-	Unimoda	2–4 μm
	Real-world road environment [87]	30 km/h braking	TSI engine EEPS	-	Unimoda	70–90 nm
		100 km/h braking	TSI engine EEPS	-	Bimodal	<10 nm, 30–60 nm
	Road simulator [88]	Nonstudded tire 70 km/h	SMPS	-	-	27 nm
		Studded tire 50/70 km/h	SMPS	-	-	44 nm

APS, aerodynamic particle sizer; EEPS, engine exhaust particle sizer; SMPS, scanning mobility particle sizer

**Fig. 6.**
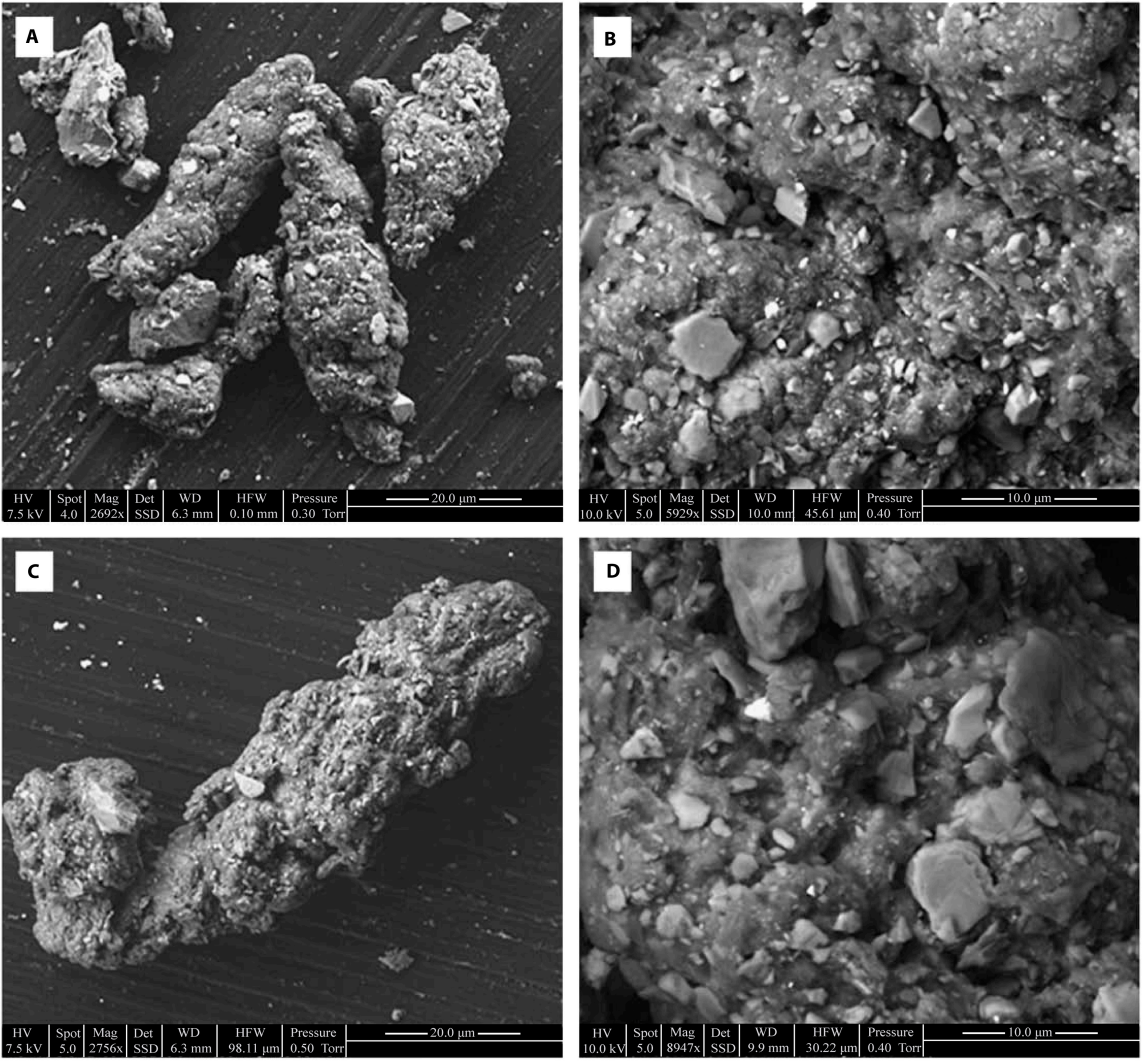
VRWMP morphology: (A and B) from real-world road environments and (C and D) from a road simulator. Reproduced from [16] with permission.

Existing studies indicate that VRWMPs are heterogeneous aggregates with a tire-rubber core encapsulated by a shell of mineral particles; consequently, their density is generally higher than that of pristine tire particles. Pure tire crumb particles have densities around 1.13 to 1.16 g/cm^3^, whereas VRWMP densities range from 1.5 to 2.2 g/cm^3^ [23,24]. Sommer et al. [25] analyzed 171 VRWMPs (10 to 18 μm in size) and found that the outer coating accounted for 10% to 50% of the particle volume. Kreider et al. [16] investigated the morphology of VRWMP collected from road environments and road simulators using scanning electron microscopy (SEM). Both sources of VRWMP exhibit a characteristic sausage-like morphology (Fig. 6), with tire-rubber particles encapsulating or embedding pavement mineral aggregate. The 2 sources show aspect ratios of 0.63 and 0.64, and roundness values of 0.84 and 0.83, respectively, further confirming their morphological similarity.

The macro-texture and aggregate gradation of road surfaces can substantially influence the initial particle size distribution of wear debris (as shown in Table 2). Experimental studies under controlled conditions have shown that the evolution of pavement texture is quantitatively coupled with the generation characteristics of wear particles. For example, wear mass loss can follow an exponential decay with a high goodness of fit. Direct contact between tires and exposed aggregates markedly increases the production of fine particles such as PM10, with coarse particles (100 to 500 μm) constituting the majority of wear debris [26].

**Table 2.** Influence of pavement macrotexture and gradation on wear particle characteristics

Research theme	Test system	Key findings	Implications for pavement effects
Long-term evolution of tire wear and pavement texture [26]	Rolling friction at 30 °C, 5.4 km/h 50% relative humidity, 0.7 MPa contact pressure	Exponential decay of pavement wear (*R*^2^ = 0.99); aggregate exposure enhances PM_10_ and coarse particles (>50%, 100–500 μm)	Texture evolution is closely coupled with the particle size distribution of VRWMP.
Inflammatory response induced by VRWMP [27]	VTI simulator particles from studded tire-asphalt pavements with granite and quartzite aggregates	Quartzite pavements induce stronger inflammatory responses; differences are linked to aggregate crush resistance.	Aggregate mineralogy and mechanical properties shape particle quantity and distribution.
Ultrafine particle emission from tire–road systems [28]	Three winter tire–pavement combinations at 70 km h^−1^ (studded/nonstudded × quartzite SMA/granite AC)	Particle size increases from nonstudded/quartzite SMA to studded/quartzite SMA and studded/granite AC (from 27 to 50 nm).	Tire type exerts the dominant influence on VRWMP and may mask pavement-related differences.

AC, asphalt concrete; SMA, stone mastic asphalt

In addition, aggregates with different mineral compositions and mechanical properties generate particles with distinct characteristics, likely due to differences in fragmentation resistance. Aggregates with a higher tendency to fracture produce greater particle numbers and altered particle features. Thus, aggregate crushing value and flakiness may influence the particle size distribution of VRWMP [27]. Tire type is another key factor controlling particle size, and its influence may even outweigh that of the pavement material itself [28]. Collectively, these findings indicate that road characteristics, including texture, aggregate mineralogy, and mechanical performance, are not passive background conditions but active determinants of VRWMP physical properties. Future work should focus on systematic quantification of these road parameters to better elucidate their roles in VRWMP generation mechanisms.

### Chemical composition

VRWMPs are heterogeneous aggregates generated through tire–road interactions during service, and their chemical composition differs markedly from that of pristine tire materials, reflecting a pronounced multi-source origin. Tire compounds are intrinsically complex composite formulations composed of rubbers, fillers, processing oils, metal reinforcements, vulcanization agents, and various functional additives (Table 2), designed to meet mechanical, durability, and processing requirements [29]. Natural rubber (NR), a linear polymer of isoprene, is widely used in high-performance tires due to its superior strength and toughness, with substantially higher contents in truck tires than in passenger car tires [26,30]. Synthetic rubbers (SRs), such as styrene butadiene rubber (SBR) and butadiene rubber (BR), serve as important complements in modern tire formulations.

Tires are a major source of organic constituents and characteristic inorganic elements in VRWMP. Zn is one of the most abundant inorganic elements associated with tire-derived particles because ZnO is widely used as an activator in rubber vulcanization, and relatively high Zn concentrations, often at the level of several thousand parts per million, have been reported in TWPs [16,31,32]. However, Zn may also be introduced from non-tire sources within road environments; therefore, it should be interpreted together with other chemical and morphological information when discussing the composition of VRWMP. Other metals, including Cu, Sb, and Cd, may also originate from tires, although their contributions are generally lower [16,33–37]. In terms of organic composition, tires contribute the majority of VRWMP organic matter, including rubber polymers, additives and their transformation products, polycyclic aromatic hydrocarbons (PAHs), and carbonaceous fractions. The complex composition and material stability of tire rubber, which underpin its extensive application in geo-engineering practices, have been systematically reviewed [38], further highlighting the diversity of organic constituents potentially contributing to VRWMP. Rubber polymers (NR, SBR, BR) constitute the core organic matrix of VRWMP [29,30,39], while additives include aromatic oils, benzothiazole-type vulcanization accelerators, antioxidants, and plasticizers [40–43]. Although PAHs are present in tires, their contribution to environmental PAH burdens is often smaller than that from secondary adsorption processes [16,35,37,42–45]. Carbon black is the main source of elemental carbon (EC), whereas polymers and additives primarily contribute to organic carbon (OC) [33,35].

**Fig. 7.**
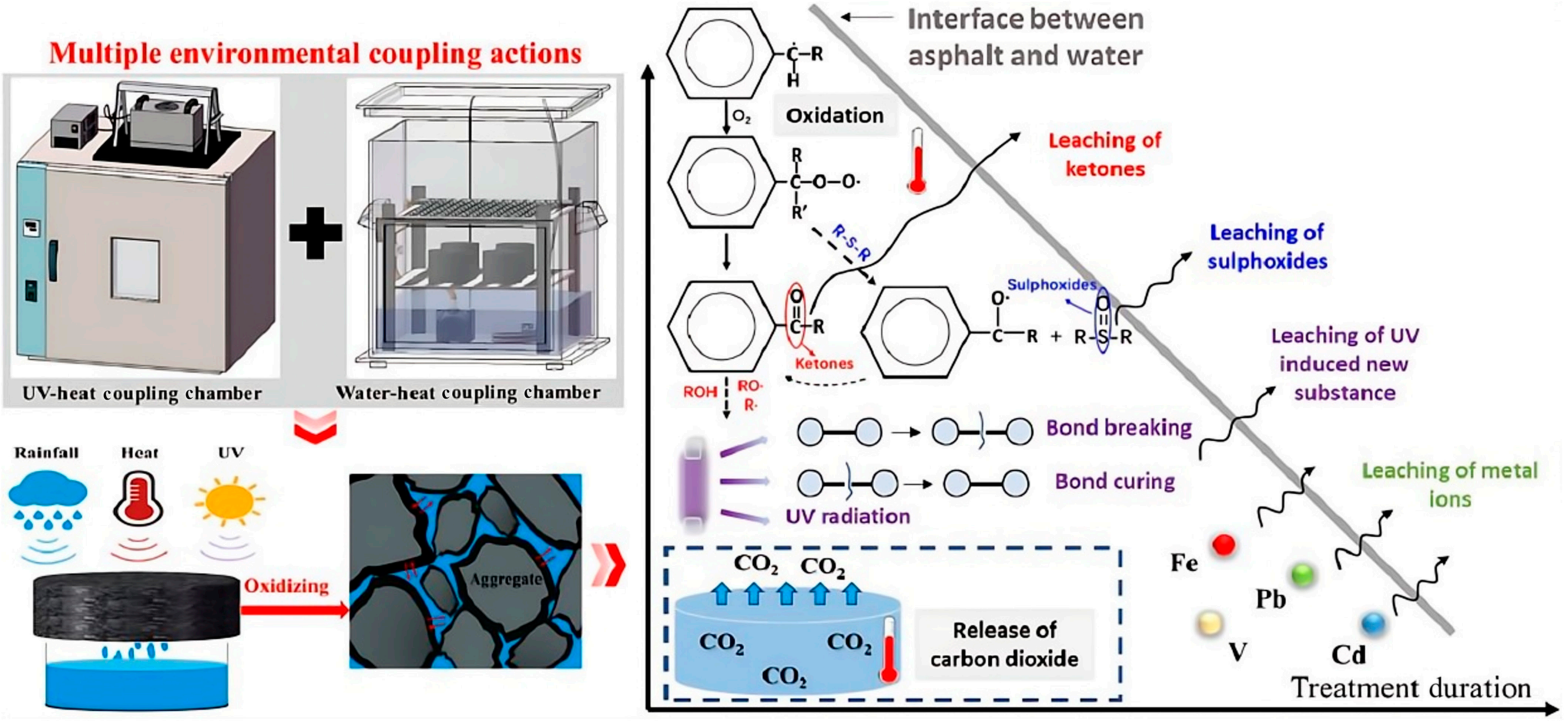
Mechanism of leaching in porous asphalt mixtures. Reproduced from [136] with permission.

VRWMPs not only are composed of tire wear but also incorporate substantial contributions from pavement materials, which have received comparatively less attention. Available studies indicate that conventional asphalt mixtures and some polyurethane-modified asphalts generally exhibit low metal leaching potential [31,46]. However, the increasing use of industrial solid wastes and chemical modifiers in road engineering may enhance the contribution of pavement materials to heavy metals and PAHs in VRWMP (Table 3). The incorporation of recycled polymeric materials is not limited to asphalt modification; polyethylene terephthalate (PET) fibers, waste plastic lightweight aggregates, and other plastic-derived components have also been introduced into cementitious construction materials [47,48]. Recycled plastic-modified asphalt serves as a conduit for exogenous polymers such as polyethylene and polypropylene, as well as associated additives including phthalate plasticizers and heavy metals (e.g., Cd, Pb, and Cr). However, direct experimental evidence on the leaching of these specific additives from recycled plastic-modified asphalt under service conditions remains limited, highlighting an important knowledge gap. Although the chemical markers of rubber asphalt—notably zinc, benzothiazoles, and 6PPD—largely overlap with tire-derived constituents, its application markedly amplifies the absolute loading of these toxic additives and may fundamentally alter their leaching dynamics, as demonstrated by long-term leaching studies on crumb rubber materials [49]. Furthermore, colored pavements utilizing inorganic pigments represent a distinct potential source of transition metals such as Cr, Ti, and Co, although systematic data under realistic service conditions remain scarce. These diverse chemical signatures underscore the heightened environmental risks of VRWMP, as interactions among multiple additives and heavy metals may intensify cumulative toxicity to roadside ecosystems, a dimension still underrepresented in current risk assessment frameworks. Notably, field evidence directly linking rubber asphalt pavements to 6PPD-Q emissions remains limited, highlighting a critical knowledge gap that necessitates dedicated investigation. Overall, the influence of pavement wear on VRWMP chemistry is governed by material type, modification strategy, environmental conditions, and aging state (Table 4 and Fig. 7). For instance, acidic conditions and prolonged contact times typically enhance metal and PAH release, whereas aging processes exhibit differential effects on binders versus mixtures (Table 5).

**Table 3.** Composition of tire materials (reproduced from [29] with permission)

Category	Content	Composition
Rubber	40–60%	Polybutadiene, styrene-butadiene, chloroprene rubber, isoprene rubber, polysulfide
Filler	20–35%	Carbon black, SiO_2_, silane
Processing oil	12–15%	Mineral oils
Steel cord	5–10%	---
Vulcanizing agent	1–2%	ZnO, S, Se, Te, thiazoles, organic peroxides, nitroso compounds
Additive	5–10%	Preservatives (halogenated nitriles), antioxidants (amines, phenols), desiccants (calcium oxide), plasticizers (aromatic and aliphatic esters), and processing aids (mineral oils, peptides).

**Table 4.** Contaminants in asphalt mixture leachate

Sample types	Analytical techniques	Metal elements
Leachate from reclaimed asphalt mixture [31]	ICP-AES	The concentrations of Cd, Cu, Cr, Ni, Pb, Zn, Hg, and Mo were all below the drinking water standard limits.
Polyurethane and cecabase-modified asphalt [46]	SW-846 test method 1312: synthetic precipitation leaching procedure	As, Cd, Cr, Cu, Fe, Mn, Pb, Zn
Asphalt mixture [142]	ICP-AES	As, Cd, Cr, Cu, Ni, Pb, Se, Zn
Leachate from porous asphalt mixture [136]	ICP-MS	V, Mn, Fe, Ni, Cu, Zn, As, Se, Cd and Pb

ICP-AES, inductively coupled plasma–atomic emission spectroscopy; ICP-MS, inductively coupled plasma–mass spectrometry

**Table 5.** Comparative findings on the chemical characteristics of VRWMP associated with pavement wear across different studies

Material types and analytical methods	Key findings (heavy metals)	Key findings (PAHs)	Primary influencing factors
CA, SA, AR, WC, BA, WCAR; ICP-AES; GC/MS; toxicity equivalent factor evaluation [142]	BA exhibits the highest heavy metal leaching, followed by WCAR, AR, RA, WC, CA, and SA.	WCAR shows the highest toxicity equivalent factor, followed by WC and BA; CA, SA, AR, and RA are close to the blank control.	Solid-waste incorporation; chemical modification
Various asphalt mixtures; ICP-MS [136]	Concentrations of V, Fe, Ni, and Zn continuously increase over time; leaching mechanisms for metals identified (Fig. 7).	—	Environmental factors (immersion duration and conditions)
Rubber-modified asphalt; DSLT standardized leaching procedure [143]	—	Total PAHs range from 0.061 to 8.322 μg/g; pyrene is the dominant PAH; larger rubber particles lead to higher PAH leaching.	Rubber content; particle size
Recycled asphalt mixtures; ICP-MS; GC/MS [144]	—	Rejuvenators increase the leaching of some PAHs; aging reduces PAH leaching.	Rejuvenator use; aging
Rubber asphalt, mortar, and mixtures under acidic and neutral conditions [145]	Low risk under neutral conditions; Cd and Zn exceed limits under acidic conditions; aging reduces metal leaching from asphalt but increases it from mortar and mixtures.	Overall low PAH release; acidic conditions increase light PAHs; aging has minimal influence.	pH; aging

AR, asphalt rubber mixture; BA, solid-waste-amended asphalt mixture; CA, neat asphalt mixture; DSLT, dynamic surface leaching test; RA, recycled asphalt mixture; SA, SBS-modified asphalt mixture; WC, warm-mix asphalt mixture; WCAR, warm-mix asphalt rubber mixture

In addition to tires and pavements, road marking materials represent a secondary source of road-related wear particles and potential microplastics [50]. Modern road markings increasingly incorporate engineered functional materials (e.g., polymer composites designed for optical or electromagnetic sensing applications) [51], which may further complicate their degradation behavior and particle emission characteristics. Under repeated tire loading, polymer binders and embedded polymethyl methacrylate (PMMA) beads in road markings can detach and fragment into fine particles [52–55]. Although road markings have been reported as a major microplastic source [56], their contribution may be overestimated relative to the continuous generation of VRWMP [52]. Thermoplastic markings with internally mixed glass beads may exhibit higher emission potential after surface bead depletion [57], but field studies indicate that their actual contributions are lower than early estimates [58]. Distinguishing marking-derived particles from VRWMP remains challenging due to limited chemical specificity; recent approaches combining automated SEM/energy-dispersive X-ray spectroscopy (EDX) with machine-learning algorithms have improved source discrimination in road dust [59].

Overall, the chemical composition of VRWMP reflects their multi-source origin: Tires primarily contribute characteristic organic compounds and Zn, pavement materials supply mineral components and additional metals and PAHs, and road markings may introduce specific polymers and trace metals. Reliable identification of VRWMP in environmental samples therefore requires multi-indicator approaches (e.g., combined S/Zn and Al/Ca/Si signatures) and source apportionment. Future studies should further quantify secondary sources and assess how emerging pavement materials reshape VRWMP composition and environmental behavior.

### VRWMP emission factor

VRWMP emission is a complex, nonlinear process governed by the coupling of tire properties (formulation, dimensions, accumulated mileage), vehicle attributes (weight, engine power, vehicle type), pavement characteristics (material, texture, environmental conditions), and operating dynamics (speed, acceleration) [29,60]. Mainstream estimation methodologies are primarily categorized into 2 approaches: (a) determining emission factors (EFs) via literature reviews and laboratory simulations, followed by estimating emissions based on the regional total annual vehicle kilometers traveled, and (b) estimating emissions based on regional tire consumption rates and annual mass loss attributed to wear.

Adopting the first approach, the International Union for Conservation of Nature established a system of empirical formulas based on mass conservation. This model not only accounts for total tire wear but also introduces environmental partition coefficients for soil, surface water, and sewage networks, attempting to quantify the fate pathways of VRWMP across multiple media [61]. Conversely, Lee et al. [62] employed the second approach to calculate VRWMP EFs for South Korea by integrating tire warranty periods, daily vehicle mileage, tire weight, and mass loss ratios while also summarizing EFs across different countries and regions (Table 6). This methodology focuses on the micro-scale loss of individual vehicles, offering a more direct calculation logic.

**Table 6.** Summary of VRWMP emission factors for different countries and regions (reproduced from [62], with permission)

Region/country	Emission factor (mg/vehicle km)			
	Passenger car	Light-duty truck	Bus	Heavy-duty truck
United Kingdom	100	-	-	-
Sweden	50	50	700	700
Germany	90	700	700	700
Urban roads in the Netherlands	132	658	415	658
Rural roads in the Netherlands	Passenger car	Light-duty truck	Bus	Heavy-duty truck
Motorways in the Netherlands	104	517	326	517
Russia	132	306	-	1,110
Denmark	132	204	-	712
Japan	1,780–2,880 cm^3^/tire	5,484 cm^3^/tire	5,484 cm^3^/tire	5,484 cm^3^/tire
Norway	132	-	-	712
South Korea	45–57	224	799	949

A critical limitation of these methodologies is their exclusive focus on tire wear contribution, often neglecting pavement abrasion due to the technical difficulty in distinguishing pavement particles from background road dust. This omission likely results in an underestimation of total VRWMP generation. To compensate, a 50:50 tire-to-pavement contribution is often assumed. European Monitoring and Evaluation Programme (EMEP)/European Environment Agency (EEA) models estimate direct PM10 pavement wear EFs for passenger, light, and heavy-duty vehicles at 5 to 10, 7.75, and 33.5 mg/vehicle km, respectively [63]; however, under studded tire usage, these values can surge to 200 to 400 mg/vehicle km [64]. Macro-evaluations using these corrected datasets indicate substantial loads, such as 75,200 to 98,400 tons/year in Germany [65] and global per capita emissions of 0.9 to 2.5 kg/year [66]. They identify freight intensity and mileage as critical drivers of total emissions.

Current research on VRWMP emissions suffers from significant uncertainty due to a predominance of indirect estimations over in situ measurements. Consequently, developing robust models based on empirical data is imperative. This is particularly urgent for China, where the massive transport network (>6 million km) and vehicle fleet (453 million by end-2024) result in tire wear contributing 53.9% of total microplastics [67]. However, there is a paucity of region-specific field data on VRWMP emissions in China, forcing most studies to rely on international datasets. Given the pronounced regional variability in VRWMP emissions, such an approach may introduce considerable uncertainties when estimating the environmental burden of VRWMP in the Chinese context.

### Technical challenges and future research prospects

It is imperative to establish a comprehensive framework for assessing the environmental impacts of VRWMP, illuminating the interactions among controlling factors and their mechanisms of interaction with the surrounding environment. At present, no standardized methodology exists for systematically characterizing the physicochemical properties of VRWMP. Owing to the diversity of tire and pavement materials and their compositional overlap, source attribution of VRWMP still relies primarily on multi-indicator approaches rather than definitive criteria. A critical knowledge gap lies in the limited investigation of wear processes associated with pavement materials and road marking coatings. The increasing diversification of pavement materials further complicates VRWMP generation pathways and release conditions, potentially exacerbating environmental risks. Although pavement materials and road markings generally account for a smaller mass fraction of VRWMP, their wear rates and contributions remain difficult to quantify. In addition, current estimates of VRWMP EFs suffer from considerable uncertainty, largely due to a paucity of representative, region-specific datasets. Most existing emission models focus predominantly on tire wear and neglect pavement wear, which may constitute a substantial proportion of total VRWMP mass, leading to systematic underestimation of overall emissions.

Future research should prioritize the development of experimental platforms capable of simulating realistic wear conditions, together with large-scale field monitoring of VRWMP to obtain primary emission data. Such efforts are critical for accurately apportioning the respective contributions of tires, pavements, and other road-related sources, thereby establishing robust emission inventories to underpin environmental risk assessment and mitigation strategies.

## VRWMP Experimental Characterization

### Method of preparation

**Fig. 8.**
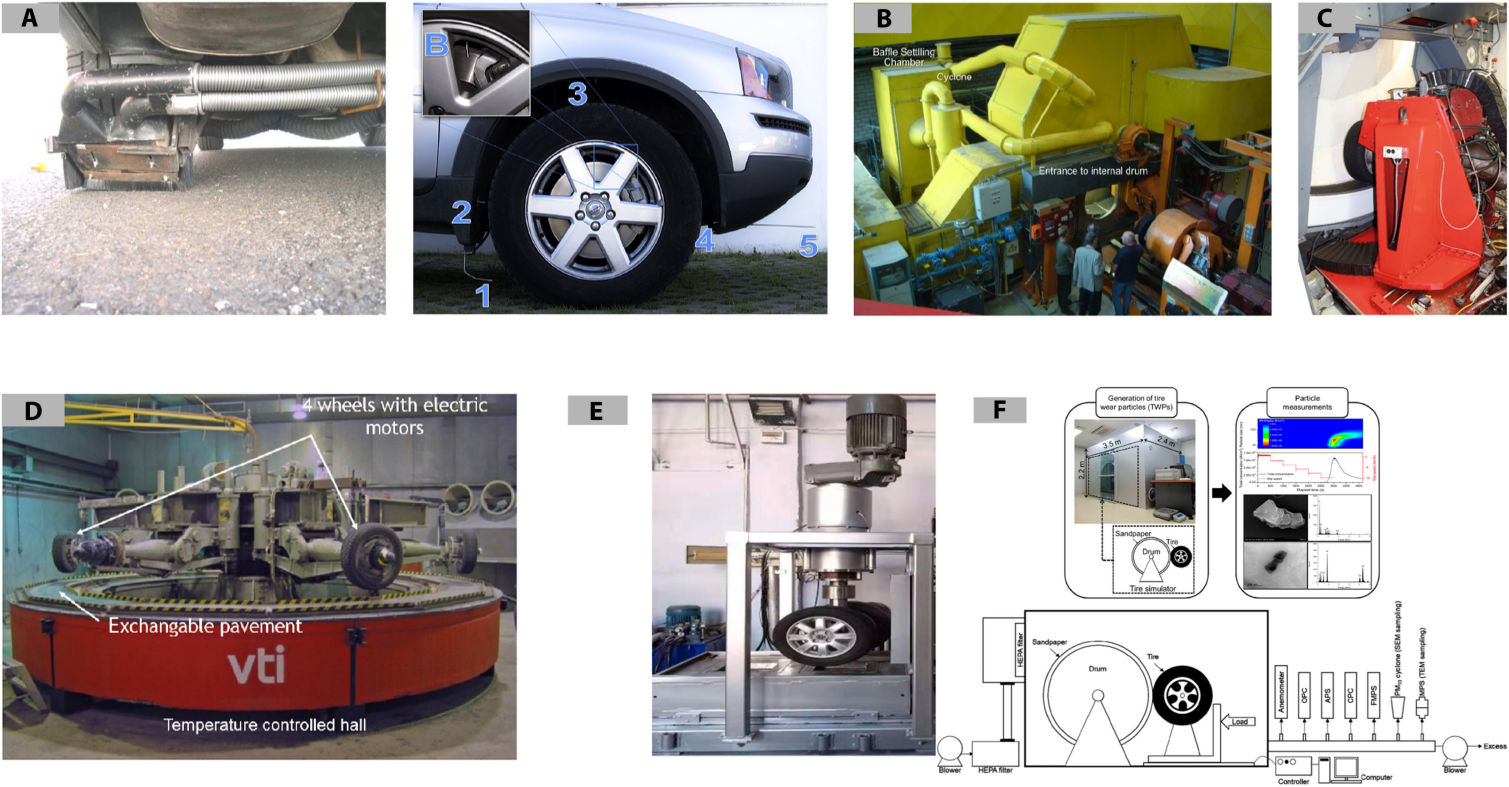
Representative VRWMP sampling and preparation equipment. (A) Road VRWMP sampling vehicle. Reproduced from [16,22] with permission. (B) BASt road simulator. Reproduced from [16] with permission. (C) Karlsruhe Institute of Technology (KIT) road simulator. Reproduced from [137] with permission. (D) Swedish VTI road simulator. Reproduced from [138] with permission. (E) Multiphysics controllable tire–road wear integrated equipment. Reproduced from [139] with permission. (F) Tire simulator. Reproduced from [140] with permission.

The current principal methods for sampling VRWMP are summarized in Table 7, and the representative equipment are given in Fig. 8. These methods can be grouped into road environment collection, real road simulator collection, and grit paper or stiff brush abrasion. Road environment collection provides the highest environmental representativeness but is susceptible to particle loss and background contamination. Real road simulators improve experimental control while retaining tire–pavement interactions, but their climatic simulation capability remains limited. Grit paper or stiff brush abrasion provides reproducible TWP samples, but it does not incorporate pavement wear and should therefore be used mainly for preliminary or comparative studies. Overall, method selection should balance environmental realism, experimental controllability, and the specific objective of the study.

**Table 7.** Summary of VRWMP sampling methods

Category	Equipment	Characteristics	Reference
Road environment collection	Road VRWMP sampling vehicle	Realistic driving conditions; nonsealed system may underestimate emissions.	[16,22,146]
	Vacuum collector	Direct surface collection; strongly affected by road contamination and sampling area.	[16]
	Polypropylene container	Suitable for deposited particles; susceptible to transport and external contamination.	[79]
Real road simulator collection	Road simulator (BASt)	Real asphalt pavement and controlled driving conditions; limited climatic simulation.	[16]
	Road simulator (KIT)	Controlled tire–road interaction; limited climatic simulation.	[137,147]
	Swedish VTI road simulator	Real tires and pavement with temperature/humidity control; high equipment complexity.	[28,138]
	Multi-physics controllable tire–road wear equipment	Multi-condition tire–road wear simulation; fine-particle collection and temperature control remain limited.	[139]
Grit paper/stiff brush abrasion collection	Tire simulator	Vacuum-based TWP generation and collection; pavement and climatic effects ignored.	[140,141]
	Grit paper/stiff brushes	Simple generation of pure TWP; low representativeness of real-driving VRWMP.	[16,147]

KIT, Karlsruhe Institute of Technology

### Characterization techniques for VRWMP

Accurate characterization of VRWMP is essential for evaluating its environmental fate, transport, and ecological risks. The analysis of VRWMP presents significant challenges due to its heterogeneous composition of tire rubber, pavement materials, and embedded road surface wear particles, coupled with the complexity of the environmental matrix. A comprehensive analytical framework that integrates sample pretreatment, physical separation, and chemical identification has been established. However, a standardized testing methodology has yet to be formulated. Developing a unified metric for VRWMP characterization would enable cross-study comparability, facilitate data harmonization, and support both industry standardization and international regulatory coordination. Importantly, such analytical methods must account for the unique physicochemical properties of VRWMP, including its complex chemical composition, broad density spectrum, and dynamic environmental behavior.

#### Pretreatment and separation methods

Given the low concentrations of VRWMP and high interference in environmental matrices (e.g., soil, sediments, water, and air), effective pretreatment is a prerequisite for accurate identification. The core objective is to maximize the removal of organic and inorganic impurities and enrich VRWMP through physical and chemical means while minimizing damage to the target particles. This process requires precise optimization, as improper treatment can lead to particle loss, morphological alteration, or chemical degradation, thereby compromising downstream analysis.

Density separation serves as the primary enrichment method, exploiting the density differential between VRWMP (1.1 to 1.9 g/cm^3^) and natural minerals (>2.0 g/cm^3^). Current strategies exhibit distinct trade-offs between universality and resolution. In resolving particle heterogeneity, a 2-step separation approach [NaCl followed by sodium polytungstate (SPT)] shows distinct advantages, enabling effective discrimination of VRWMP fractions with different degrees of mineral encapsulation [68]. In contrast, a single-step high-density SPT method (e.g., 1.9 or 2.2 g/cm^3^) allows efficient recovery of the majority of VRWMP [69,70]. Furthermore, refined gradient density separation (1.20 to 1.70 g/cm^3^) enables the precise fractionation of VRWMP components, providing methodological support for investigating their differential environmental behaviors [71].

Innovation in chemical purification focuses on material compatibility and the specific removal of pavement-derived interferences. Alkaline digestion, such as with NaOH, shows better compatibility compared to strong oxidants like H_2_O_2_ and NaOCl. It can effectively remove organic matter without damaging the elastic structure of VRWMP [68]. These chemical steps are complemented by standardized sieving (<1 mm or <500 μm) and multi-stage filtration—using 1.0-μm filters for particle collection and 0.22-μm filters for purifying extracts prior to ultra-performance liquid chromatography (UPLC)/gas chromatography mass spectrometry (GC-MS) analysis [69,71]. Notably, to address interference from asphalt particles in road dust, chloroform extraction has been developed to selectively eliminate bituminous components, significantly enhancing identification accuracy by removing visual obstructions while preserving VRWMP morphology [72]. Additionally, high-speed centrifugation facilitates the concentration of VRWMP from aqueous samples or digestates, serving as a fundamental solid–liquid separation technique where parameter optimization is critical for maximizing recovery. Collectively, these methods constitute an integrated analytical workflow for the precise isolation of VRWMP from complex environmental matrices.

#### Identification and characterization techniques

Given the inherent heterogeneity of VRWMP, single-analytical techniques are often insufficient for conclusive identification. Consequently, a multi-methodological approach has emerged as the consensus, employing a weight-of-evidence strategy that corroborates physical morphology with chemical composition to enhance reliability.

While optical microscopy can be used as a preliminary tool for particle counting and aspect ratio analysis, it lacks chemical specificity [71]. In contrast, SEM-EDS (energy-dispersive spectroscopy) combines high-resolution morphological observation with elemental composition analysis, thereby enabling more reliable identification and characterization of VRWMP. By identifying characteristic wear morphologies and elemental signatures (tire: S/Zn; pavement: Si/Ca/Al), specifically the co-existence patterns of S with Zn/Na, SEM-EDS provides critical evidence for VRWMP mineral encrustation [68,70,73]. Additionally, the heat resistance test offers a robust, engineering-specific auxiliary method; exploiting the thermal stability gap between vulcanized rubber and thermoplastic bitumen, it effectively eliminates bituminous interference at 150 °C [68].

Chemical analysis has evolved from qualitative detection to high-throughput and high-precision quantification. Vibrational spectroscopy techniques, including Fourier transform infrared spectroscopy (FTIR) and Raman spectroscopy, together with automated laser direct infrared imaging, enable high-throughput chemical mapping by recognizing the spectral fingerprints of SRs such as SBR and BR [68]. For absolute quantification, thermal analysis coupled with mass spectrometry, particularly pyrolysis gas chromatography/mass spectrometry (py-GC/MS) and thermal extraction/desorption–gas chromatography–mass spectrometry (TED-GC/MS), is widely regarded as the gold standard. These techniques rely on characteristic pyrolysis products, such as vinylcyclohexene derived from SBR, isoprene from NR, and rubber-related additives including 6PPD and benzothiazoles, allowing highly specific identification in complex environmental matrices [68,71]. Surface-sensitive techniques such as time-of-flight secondary ion mass spectrometry (TOF-SIMS) provide complementary micro-interfacial evidence by detecting diagnostic organic fragments, for example, C_7_H_7_^+^ [70]. In addition, elemental markers, including Zn and chemically bound sulfur, as well as organic indicators such as OHBT and ABT in aquatic systems, have been applied in both quantitative and indicative studies of VRWMP [69,74].

**Fig. 9.**
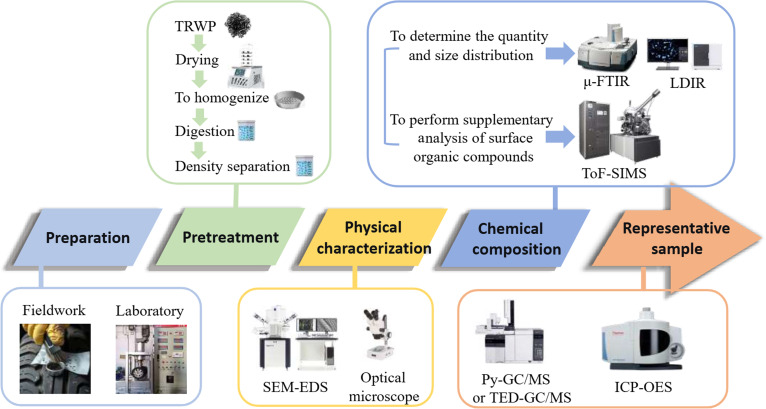
Standardized VRWMP characterization process.

Based on current practices, a maturing standardized multi-step workflow (Fig. 9) has been established, integrating morphological confirmation with chemical quantification. The protocol proceeds as follows: (a) enrichment: samples undergo drying, NaOH digestion, and density separation; (b) screening: physical exclusion of pavement interferences via SEM-EDS and heat resistance testing; (c) confirmation and quantification: chemical imaging via μ-FTIR/laser-induced direct infrared imaging (LDIR) and precise quantification using py-GC/MS markers, with TOF-SIMS providing supplementary surface data for complex samples. This multi-dimensional integration establishes a viable paradigm for resolving cross-study data comparability issues.

### Technical challenges and outlook for standardization processes

Existing VRWMP sampling and preparation methods differ substantially in environmental representativeness, experimental controllability, and particle recovery efficiency. These methodological differences limit the comparability of particle size distribution, morphology, chemical composition, and EFs across studies. Therefore, standardized protocols for sampling design, particle recovery, sample pretreatment, analytical identification, and data reporting are urgently needed. In addition, reliable detection of submicrometer VRWMP and robust quantitative thermal analysis remain constrained by sample recovery loss, matrix interference, detection limits, and the lack of standardized reference materials. Although manual single-particle analysis remains applicable, it is time-consuming and prone to reproducibility limitations, particularly for complex environmental and biological matrices. Future studies should combine standardized experimental workflows with automated image analysis, spectral identification, and machine learning-assisted data integration to improve the efficiency, reproducibility, and comparability of VRWMP characterization.

Recent advances in artificial intelligence, deep learning, and large-scale models may further support the standardization of VRWMP analysis. Deep neural networks can assist in the automated recognition and classification of VRWMP from SEM images, optical images, and spectral datasets, while machine-learning workflows can integrate heterogeneous information, including morphology, elemental composition, spectral signatures, and thermal decomposition products. In addition, large-scale models trained on well-annotated datasets may help optimize pretreatment parameters, reduce operator-dependent bias, and improve the prediction of VRWMP concentrations and compositional profiles when reference materials are incomplete. Therefore, coupling AI-based data analysis with standardized experimental protocols represents a promising direction for improving the accuracy, efficiency, and cross-study comparability of VRWMP characterization.

## VRWMP Migration Mechanism

**Fig. 10.**
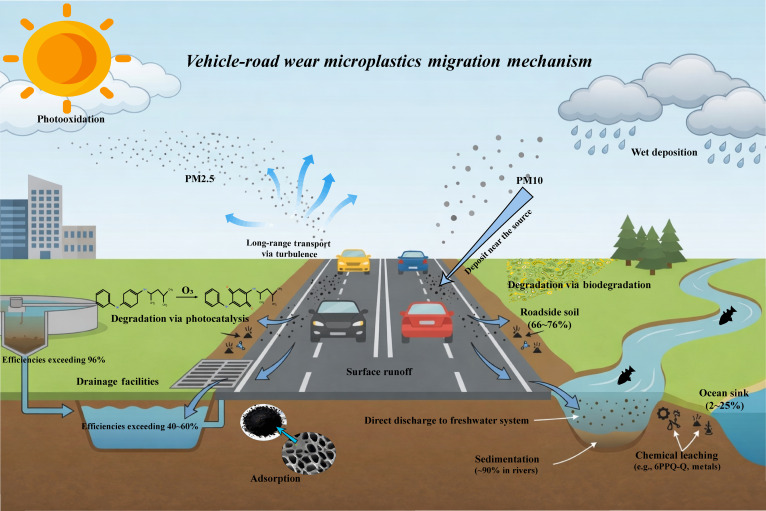
The migration mechanism in the environment of VRWMP. The generation of VRWMP from tire–pavement interactions on road surfaces, followed by their partitioning into 3 primary pathways: atmospheric dispersion, surface runoff, and roadside soil deposition.

VRWMP undergoes complex physical transport and chemical transformation within road systems and across environmental media driven by external forces such as gravity, wind, and water flow [75], as illustrated in Fig. 10. These processes are governed by the combined influences of particle physicochemical properties, ambient environmental conditions, and interactions with surrounding media, which collectively determine its environmental fate and ecological risks. A comprehensive understanding of VRWMP migration mechanisms therefore provides the scientific basis for assessing its environmental impacts and developing effective mitigation and control strategies.

### Migration patterns and mechanisms within road systems

Within the road system, VRWMP undergo generation, initial partitioning, and early-stage transformation; transport behaviors during this phase directly determine the flux and physicochemical speciation of particles dispersing into the surrounding environment.

#### VRWMP markers

Accurate quantification of VRWMP distribution within road systems and the environment relies on highly specific and stable chemical markers. Identifying suitable markers constitutes the primary task for quantitatively analyzing VRWMP distribution in environmental media. Current research on VRWMP is predominantly tire-oriented, and therefore, tracers are primarily selected from rubber polymers and tire additive components. The selected tracers should meet the following criteria: ubiquitous presence in all tires, resistance to leaching or transformation from particles, high specificity to tires, significantly higher concentrations in tires than in environmental background levels, and ease of analytical detection [29].

Early tracer studies primarily focused on bulk tire components. Rubber polymers, zinc, and high-carbon n-alkanes (>C35) were commonly employed as generic markers; however, their validity is compromised by non-tire source interference (e.g., galvanized infrastructure and engine oil) and detection limit constraints [33,76,77]. Subsequent research shifted toward highly specific organic additives. While benzothiazoles are widely utilized, their semi-volatility, high polarity, leachability, and non-unique origins limit quantitative accuracy [29,45,76,78]. More recently, attention has turned to stable vulcanizing agents like 1,3-diphenylguanidine (DPG) and antioxidants such as 6-PPD. Notably, the oxidation product 6-PPDQ has emerged as a dual-purpose marker for both source tracking and ecological risk assessment due to its hydrophobicity and demonstrated aquatic toxicity [78–80].

Crucially, these single-marker approaches inherently characterize only tire wear, systematically neglecting the contribution of pavement materials within VRWMP aggregates [29]. To address this limitation, combined analysis via py-GC/MS has become the leading trend. By simultaneously detecting characteristic pyrolysis products of both tire (SBR + BR) and pavement (SBS) matrices, this method achieves holistic quantification of VRWMP. Demonstrating high recovery rates of 83% to 104% even in complex media like snow [81,82], py-GC/MS confirms the tire–road interaction nature of these particles. Consequently, developing joint quantification techniques based on material pyrolysis fingerprints, rather than seeking novel single chemical markers, represents the future mainstream direction for precise VRWMP source apportionment.

In summary, VRWMP represents a product of tire–road interaction and therefore its quantification must consider the composite contributions of both materials. Accordingly, the selection of VRWMP markers should reflect the material composition of both tires and road surfaces. Compared with the discovery of new molecular markers, pyrolytic analysis has yielded substantial progress in VRWMP quantification. With continued advancements in pyrolytic analytical technologies, the quantitative characterization of VRWMP is expected to achieve even greater accuracy and reliability in future environmental assessments.

#### Physical migration

The physical migration of VRWMP within road systems results from the multi-physics coupling of gravity, wind, vehicle-induced turbulence, and surface runoff. Consequently, the fate of these particles is governed by the interplay between particle size fractionation, pavement texture retention, and drainage infrastructure configuration. Upon generation, larger particles rapidly settle via gravity, whereas finer fractions remain suspended, exhibiting prolonged atmospheric residence times.

At the air–road interface, VRWMP frequently undergoes agglomeration with non-exhaust emissions and mineral dusts. This process follows a distinct size-dependent trajectory: Particles <10 μm are prone to resuspension and long-range transport via turbulence, while coarse fractions (10 to 100 μm) tend to deposit near the source, particularly in zones of low airflow velocity [50,83,84]. In contrast to open roads where concentrations typically decay with distance, semi-enclosed environments like tunnels exhibit high accumulation due to restricted dispersion [83]. Although the atmospheric presence of VRWMP is well-documented, the specific dynamic migration mechanisms remain under-characterized. Addressing this gap is critical given the direct inhalation risks posed by airborne VRWMP.

At the pavement–runoff interface, pavement texture retention plays a dominant role, trapping approximately 58.3% of particles within surface voids. The nonretained fraction is mobilized toward the roadside, driven primarily by rainfall runoff and street cleaning operations [50]. While experimental quantification of roadside wash-off remains limited, modeling by Unice et al. [85] estimates that 25% to 75% of VRWMP enters the roadside environment via runoff, whereas Sieber et al. [86] suggest a total environmental release proportion of 73.6%.

The terminal fate of VRWMP exhibits strong infrastructure dependency. Rural roads lacking drainage facilitate direct discharge into roadside soils and adjacent water bodies. Conversely, urban systems routed to wastewater treatment plants achieve removal efficiencies exceeding 96% [87,88]. However, highway stormwater ponds demonstrate lower retention rates of 40% to 60%, posing higher pollution risks from untreated runoff [85]. Once in aquatic systems, migration is dictated by particle properties and hydrodynamics. Due to their high density (>1.2 g/cm^3^), which exceeds that of pristine tire rubber, approximately 90% of VRWMP settles in riverbed sediments, with only a negligible fraction, about 2%, transported to marine environments [85].

In summary, VRWMP physical migration is fundamentally determined by particle properties and external forces. Current migration models, predominantly derived from foreign datasets, may not adequately capture the regional heterogeneity of VRWMP distribution in China. Given the country’s vast territory, complex climatic zones, and significant regional variations in traffic conditions, a single generalized model is insufficient for accurate nationwide prediction. Consequently, there is an urgent need for localized research to establish VRWMP migration models tailored to these specific contexts. In this regard, leveraging emerging AI technologies to construct intelligent prediction models capable of addressing multi-factor coupling represents a promising research frontier.

#### Chemical migration

Chemical migration is a key process governing the environmental behavior of VRWMP. Owing to their composite nature, VRWMPs are not chemically inert but continuously interact with surrounding media during environmental transport through adsorption, degradation, and leaching. These processes collectively control the migration, transformation, and release of associated contaminants. Therefore, investigating the chemical migration of VRWMP, particularly the influence of pavement materials and structural design, is essential for understanding their environmental fate.

Adsorption determines the contaminant loading of VRWMP and reflects their intrinsic material heterogeneity. As composites consisting of tire rubber and pavement-derived components, VRWMPs exhibit pronounced component-dependent adsorption behavior [89,90]. Carbon black primarily governs contaminant adsorption at particle surfaces, whereas the polymer matrix controls the diffusion of adsorbed compounds into the particle interior [91,92]. Adsorption capacity increases with carbon black content but decreases with higher crosslinking density and is further regulated by particle size, surface charge, and morphology [89,92]. VRWMP show a stronger affinity for hydrophobic organic contaminants than for heavy metals, while metals such as Cd and Zn are retained less stably and are highly sensitive to salinity changes [93,94]. This suggests that winter deicing practices may promote metal desorption and secondary environmental release [95]. Notably, adsorption by VRWMP cannot be attributed solely to tire rubber, as asphalt also exhibits substantial adsorption potential for both organic and inorganic contaminants, with performance varying with temperature and humidity. Thus, VRWMP adsorption is intrinsically linked to pavement material composition and structure.

Degradation governs the chemical evolution of VRWMP but proceeds slowly under realistic environmental conditions. Different polymers follow distinct degradation pathways: BR and SBR are prone to photo-oxidative chain scission, whereas NR and isoprene-based rubbers, characterized by cis-1,4-isoprene structures, show higher biodegradation potential [19]. Under optimized conditions, specific microbial consortia can achieve substantial mineralization (40% to 50%) within weeks, as shown in Table 8. However, in natural environments, VRWMP degradation rates of 0.15% per day remain very low, far below physical removal rates of 0.67% per day, largely due to the shielding effect of carbon black and the intrinsic stability of polymers [96]. Although degradation contributes little to mass reduction, its role in modulating chemical hazards is significant. Under traffic-induced thermal and photo-oxidative conditions, 6PPD can be transformed into the highly toxic 6PPD-Q, a reaction strongly controlled by ozone concentration and elevated temperatures (>80 °C) [97].

**Table 8.** Comparison of typical VRWMP degradation pathways, influencing factors, and experimental performance

Degradation type	Key drivers	Key experimental findings and degradation rates	Environmental constraints and limitations
Biodegradation [125,148–150]	Nocardia sp., Rhodococcus sp.*,* Pseudomonas, etc.	8-week mineralization rate of 40–50%; NR degradation (55.3%) superior to SR (45.9%)	Effectively degrades plasticizers and paraffin fatty acids; performance is limited by isolated inoculum and static temperature conditions
Chemical transformation [97]	Traffic-induced coupled thermal and photo-oxidation	Degradation of 6PPD into 6PPD-Q	Ozone and high temperature (>80 °C) promote transformation and increase toxicity
Photo/thermal oxidation [96,151]	UV radiation, high temp (>60 °C), ozone, mechanical stress	Thermal and UV aging induce mineral de-aggregation, reduce density, and deplete extractable organics; mechanical stress decreases particle roundness	Primarily abiotic process; degradation rates are constrained by carbon black shielding

SR, synthetic rubber; UV, ultraviolet

Leaching represents the primary pathway by which VRWMPs cause direct environmental exposure. Under hydrodynamic conditions such as rainfall runoff, VRWMPs release heavy metals and a range of organic additives, including Zn, 6PPD-Q, DPG, aniline compounds, benzothiazoles, and hexamethoxymethyl melamine (HMMM) [98,99]. Field observations indicate that benzothiazoles in Pearl River Delta River runoff and 6PPD-Q in urban road dust in Tokyo are predominantly derived from VRWMP, with release occurring rapidly on hourly timescales [97,98]. Leaching behavior is strongly particle size dependent, with smaller particles exhibiting higher release potential due to larger specific surface areas, and cumulative release increasing with contact time [23,97]. Environmental controls are compound-specific: Acidic conditions enhance inorganic release, while higher salinity and pH generally suppress Zn leaching; however, in seawater systems, light exposure promotes Zn release [100]. Additional factors such as temperature, turbulence, ultraviolet radiation, and CO_2_ further modify leaching fluxes and chemical composition, often amplifying toxic effects on aquatic organisms [101].

Overall, the chemical migration of VRWMP is governed by the coupled effects of material composition and environmental drivers. The strong dependence of adsorption and leaching on pavement materials suggests that emerging road technologies, such as rubber-modified asphalt and waste-incorporated asphalt mixtures, may substantially reshape VRWMP chemical emission profiles. From a clean-materials perspective, integrating chemical migration mechanisms into tire formulation and pavement material design is critical for source-oriented environmental risk control. Consequently, elucidating VRWMP leaching mechanisms, particularly through molecular dynamics simulations to resolve micro-scale processes, represents a promising research direction, providing a theoretical basis for designing environmentally benign tires and pavement materials and enabling leaching risk mitigation at the source.

### Distribution trends from road systems to other environmental media

Model-based studies provide quantitative insights into the distribution of VRWMP from road systems to other environmental compartments. Overall, the environmental fate of VRWMP exhibits a clear pattern, with roadside soils and freshwater systems serving as the primary sinks. As a significant source of microplastics released unintentionally into the environment, the fraction of VRWMP in the atmosphere is extremely low, approximately 2% to 10%. Conversely, the overwhelming majority, approximately 49% to 76%, accumulates in roadside soils through dry and wet deposition, establishing soil as the primary environmental sink [21,65,76,84]. At the watershed scale, particle fate exhibits strong density dependence. The mass balance model constructed by Unice et al. [85] demonstrates that although approximately 49% of VRWMP enters the water cycle via runoff, freshwater systems exhibit a retention efficiency as high as 90%, which is primarily achieved via sedimentation in riverbeds. Consequently, the flux ultimately transported to the ocean is negligible, amounting to only about 2%.

The configuration of road infrastructure significantly modulates the input flux of VRWMP into water bodies. Investigations of German motorways indicate that 90% of emissions are deposited in the near-source region of the road surface or roadside [29]. Further model estimates reveal that in scenarios employing comprehensive drainage facilities, the proportion of VRWMP entering receiving waters is controlled between 6% and 23%; however, in conditions lacking such facilities, this ratio rises significantly to 13% to 45% [85].

Existing research has preliminarily elucidated the distribution trends of VRWMP within road systems through modeling, identifying soil as the main accumulation reservoir and water bodies as critical migration pathways. These studies clarify the pivotal roles of particle size and road drainage infrastructure, identifying particle size and density as the sensitive parameters controlling the output flux to estuaries. Although current research has clarified these common mechanisms, the parameters for distribution models are largely derived from foreign datasets, which introduces significant regional limitations and uncertainty. Direct application to China may yield substantial discrepancies. Therefore, there is an urgent need to construct environmental fate models adapted to China’s complex road and hydrological characteristics based on local empirical data, thereby providing a robust basis for precise pollution control.

### Technical challenges and future research prospects

Understanding the migration of VRWMP is essential for evaluating their environmental fate and ecological risks. After generation on road surfaces, VRWMP can enter air, soil, runoff, drainage systems, sediments, snow, and ice through resuspension, deposition, stormwater transport, and snowmelt processes [19]. Their migration is affected by particle properties, rainfall, wind, traffic disturbance, pavement characteristics, and drainage conditions [69,85,102]. However, the relative contribution of different pathways remains difficult to quantify because VRWMP is a heterogeneous mixture from tires, pavement materials, road markings, deposited dust, and other road-associated sources. Current identification methods still have limitations. Many studies rely on tire-related markers, such as rubber polymers, Zn, benzothiazoles, and tire additives, which may not fully represent the road-derived fraction of VRWMP [29]. Future studies should develop multi-marker approaches combining rubber-specific organic markers, road-material signatures, elemental profiles, morphology, and thermal decomposition products. Pyrolysis-based methods, particularly py-GC/MS, are promising for complex environmental samples, but standardization in marker selection, calibration, and sample pretreatment is still needed [103,104].

During migration, VRWMP may undergo fragmentation, oxidation, aging, pollutant adsorption, additive leaching, and microbial degradation [104,105]. These processes change particle size, surface properties, chemical composition, mobility, toxicity, and persistence. Therefore, VRWMP migration should be regarded as a coupled process involving transport, transformation, and contaminant interaction. Future research should integrate field monitoring, controlled experiments, traffic data, meteorological records, pavement information, and drainage characteristics to develop multi-media migration models. Data-driven methods may further help identify controlling factors and predict VRWMP transport and environmental fate under real road conditions.

## VRWMP Environmental Impact Assessment

**Fig. 11.**
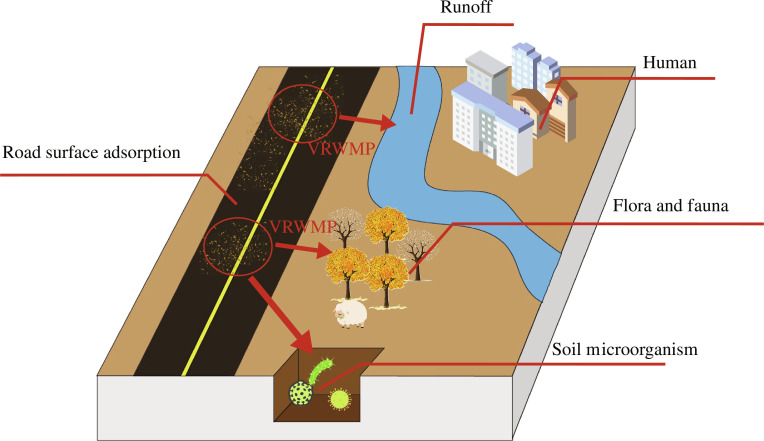
The environmental impact of VRWMP.

As a non-exhaust particulate matter generated from road transport systems, VRWMP exerts environmental impacts throughout the entire lifecycle of highway construction operation and maintenance. Current research on VRWMP’s environmental impact predominantly focuses on the particles themselves and their leachate, assessing potential ecological risks through ecotoxicological effects on soil flora and fauna, micro-organisms, aquatic organisms, and human health. However few studies consider the environmental impact of the road infrastructure projects themselves. From a road engineering perspective, the environmental impact of VRWMP can be systematically categorized into 3 levels: the ecological environment, water environments, and the broader social environment along the project route surface, as illustrated in Fig. 11.

### Environmental impact assessment

Road construction and operation permanently alter land use along transport corridors and create continuous zones of non-point-source pollution. Among the environmental compartments receiving tire- and road-derived particles, soil has been recognized as a major sink. Previous studies have estimated that approximately 67% of emitted TWPs may ultimately enter soil, with reported concentrations ranging from 100 to 117,000 mg/kg [76]. Therefore, roadside soil represents a critical medium for evaluating the environmental impacts of VRWMP.

To provide a structured summary of the assessment methods and reported effects, Table 9 summarizes soil-centered assessment dimensions, models, endpoints, and environmental impacts of VRWMP-related particles. The ecological effects of VRWMP in soil ecosystems involve multiple levels of biological organization. At the soil-function level, VRWMP-related particles may alter soil physicochemical properties, OC cycling, nutrient turnover, and microbial activity [106–109]. At the organism level, soil invertebrates, including *Folsomia candida*, *Enchytraeus crypticus*, *Eisenia fetida*, and *Tenebrio molitor*, have been widely used to evaluate particle-induced toxicity through endpoints such as survival, growth, reproduction, oxidative stress, immune response, digestive biomarkers, and bioaccumulation [34,108,110]. For example, micronized car TWPs can induce heavy metal bioaccumulation and oxidative stress in *E. fetida* [111]. Tread rubber and road dust particles can affect stress, immunity, and digestive biomarkers in *T. molitor* larvae [112]. Road dust particles may also induce sublethal physiological effects in *T. molitor*, indicating that toxicity assessment should not rely only on mortality or reproduction endpoints [113]. In addition, TRWP can act as carriers of metals and rare earth elements, contributing to trace-element bioaccumulation in soil organisms [114]. At the sub-organism level, gut microbiota and immune-related responses may be more sensitive than traditional endpoints such as mortality or reproduction. Exposure-induced dysbiosis can promote opportunistic pathogens and impair host physiological regulation, indicating that microbiome-level endpoints are important for understanding the ecological effects of VRWMP-related particles [34,110]. In addition, atmospheric deposition and foliar accumulation of traffic-related microplastics in crops suggest that VRWMP may enter roadside agricultural systems and potentially contribute to trophic transfer [115,116].

**Table 9.** Soil-centered assessment dimensions, models, endpoints, and reported effects of VRWMP-related particles

Assessment dimension	Assessment model/method	Endpoint	Main effects	Reference
Soil ecosystem function	Soil physicochemical and microbial assessment	Soil structure; organic carbon cycling; nutrient turnover; microbial activity	VRWMP-related particles may alter soil structure, organic carbon cycling, nutrient turnover, and microbial activity.	[29,102–104]
Soil fauna toxicity	Soil invertebrate exposure model	Survival; growth; reproduction; oxidative stress; immune and digestive biomarkers	Exposure to tire wear particles, road dust particles, and related particulate pollutants can affect survival, growth, reproduction, oxidative stress, immunity, digestive biomarkers, and physiological status.	[34,103–107]
Carrier effect and bioaccumulation	Elemental bioaccumulation analysis	Metals; rare earth elements; trace-element accumulation	TRWP can act as carriers of metals and rare earth elements, contributing to trace-element bioaccumulation in soil organisms.	[108]
Microbiome-level response	Gut microbiota analysis	Gut microbiota composition; dysbiosis; host physiological regulation	Gut microbiota dysbiosis may be a sensitive sub-organism endpoint and may impair host physiological regulation.	[34,104]
Roadside vegetation and trophic transfer	Foliar absorption and plant accumulation assessment	Foliar retention; plant accumulation; potential food-chain transfer	Atmospheric microplastics can accumulate on or enter plant leaves, indicating a potential pathway into roadside agricultural systems.	[109,110]
Material-specific toxicity	In vitro inflammatory response model	Cellular inflammatory response; material-dependent toxicity	Pavement aggregates and mineral components can modulate the toxicity of tire–pavement wear particles.	[111]

However, current environmental impact assessments of VRWMP still show a clear mismatch between laboratory toxicity tests and field exposure conditions. Most studies rely on short-term, single-species exposure models and simplified particle systems, which limits their ability to capture long-term ecological effects, community-level cascades, and multi-stressor interactions under real road environments. Evidence from other soil particulate pollutants, such as bituminous coal dust, also shows that subchronic particle exposure can impair reproduction in soil invertebrates, further supporting the need to include chronic and functional endpoints in soil particle risk assessment [117]. More importantly, many toxicity studies focus on pristine tire particles or TWPs, while the contribution of pavement engineering materials, road dust matrices, and mineral components in VRWMP aggregates remains insufficiently considered. Since pavement aggregates and mineral components can significantly modulate particle toxicity [118], and TRWP can act as carriers of metals and rare earth elements that accumulate in soil organisms [114], neglecting the pavement-derived fraction may lead to biased estimates of the actual ecological risks associated with road infrastructure.

### Surface water environmental impact assessment

Road runoff serves as the primary pathway for the entry of VRWMP into aquatic environments. During rainfall or snowmelt events, VRWMP deposited on road surfaces are mobilized and washed off into receiving water bodies [19]. Numerous studies have detected traces of VRWMP in surface waters, indicating its ongoing impact on aquatic ecosystems. Barber et al. [119] were the first to report VRWMP concentrations in residual solids from river samples, while Kovochich et al. [120] conducted VRWMP detection across various river sections, developing a particle analysis method to determine particle size distribution in urban river sediment samples. Research has shown that VRWMP affects aquatic organisms through both physical and chemical pathways. Physical effects include ingestion, leading to digestive tract blockages, internal injury, or false satiety [121], as well as physical interference with feeding, respiration, or locomotion [122]. The chemical effects of VRWMP are primarily linked to zinc and organic compounds present in leachate, which can induce oxidative stress, cause DNA damage, and impair reproduction, growth, and survival in aquatic organisms [25,29,123]. Furthermore, toxicity identification evaluation has been incorporated into recent research methodologies, successfully identifying zinc and aniline as the most probable candidate toxicants [124], thereby providing crucial targets for source control and mitigation. The primary current challenge lies in assessing environmental relevance. Acute toxicity tests have generally indicated low toxicity for VRWMP [median effective concentration (EC_50_) > 10,000 mg/l]; however, these results were obtained under idealized laboratory conditions. In real-world scenarios, pavements are subjected to elevated temperatures in summer and exposure to de-icing salts during snowmelt periods. These “harsh conditions” have been shown to potentially enhance the release of toxic constituents from VRWMP [84,125].

Although current design standards for pavement materials and drainage systems fail to consider the exacerbating effects of extreme environmental factors on VRWMP leaching, this oversight may lead to an underestimation of actual environmental risks. Particularly within the context of climate change and the increasing frequency of extreme weather events, this assessment bias warrants greater attention from the road engineering and environmental management communities.

### Social environmental impact assessment

The health risks posed by VRWMP to roadside residents and workers constitute a central socioenvironmental concern regarding road infrastructure. Although recent findings in *Nature* reveal that micro/nanoplastics accumulate in the human brain at concentrations significantly higher (approximately 30-fold) than in the liver or kidneys, increasing over time [126], understanding specific VRWMP-induced health effects remains in its infancy due to the particles’ pronounced physicochemical heterogeneity.

While microplastics generally enter the body via ingestion, inhalation represents the dominant exposure pathway for VRWMP. Fine particulates (<2.5 μm) can penetrate respiratory barriers and persist in the lungs [127,128]. In vitro assays confirm that VRWMP induces DNA damage and inflammatory responses in macrophages and pulmonary epithelial cells [129]. Notably, pavement mineralogy modulates toxicity; VRWMP generated on granite pavements elicits significantly higher cytokine release than that from quartzite surfaces, highlighting the critical toxicological contribution of road materials [118]. However, a discrepancy exists between potential toxicity and actual risk: The NOAEL (no observed adverse effect level) established in rats (112 μg/m^3^) far exceeds ambient VRWMP concentrations (<0.1 μg/m^3^), suggesting that acute inhalation risks at current environmental levels may be low, although this requires further validation [130].

Dermal exposure is considered a secondary pathway; due to the stratum corneum barrier, direct penetration is restricted to nanoplastics (<100 nm), and absorption mechanisms remain ill-defined [131]. In addition to respiratory effects, cardiovascular risks are well-documented. Blood analyses indicate that plastic particles are bioavailable and exhibit slower clearance rates from organs compared to blood absorption [132]. Mechanistically, the leaching of water-soluble Zn and Cu from tread particles induces cardiac oxidative stress [133]. Furthermore, epidemiological studies link occupational exposure in highway patrol officers to impaired heart rate variability and induced pro-inflammatory and pro-thrombotic responses [134].

### Technical challenges and future research prospects

The environmental impact assessment methodology for VRWMP must be grounded in realistic exposure scenarios. However, existing research has largely neglected the influence of road construction factors, often overlooking the contribution of pavement materials to the overall toxicity of VRWMP. In recent years, sustainable construction practices incorporating recycled and waste materials within a circular economy framework have been increasingly adopted in road engineering [135], introducing new material compositions and surface properties that may further influence VRWMP generation, transformation, and toxicity. Current studies predominantly rely on single-species tests conducted under controlled laboratory conditions, which fail to capture the complexity of real-world ecosystems. During atmospheric dispersion, VRWMP can adversely affect flora and fauna, particularly in roadside farmlands, rivers, and aquatic environments, and may also enter the human body indirectly through the food chain.

Future investigations should broaden their scope to include microbial communities and soil ecosystems along actual roadways, moving beyond single-species assessments. Long-term field monitoring and toxicity testing are essential for developing a dynamic ecological risk assessment framework. The inherently complex physicochemical properties of VRWMP make it challenging to establish clear causal relationships between exposure and human health outcomes. Additionally, research on the accumulation, migration, and chronic effects of VRWMP within human tissues and organs is extremely limited, which hampers comprehensive risk evaluation. Future research should deepen the study of human health exposure science and toxicology, and focus on developing highly sensitive detection methods to track the migration and toxicological effects of nanoscale VRWMP after they enter the human body via respiratory tract and skin exposure. It should also explore the body burden thresholds within the organism to provide a solid scientific basis for public health protection.

Compared with recent VRWMP-focused reviews that mainly synthesize environmental occurrence, fate/transport, and ecotoxicity evidence for “tire-derived particles” or broader non-exhaust PM mixtures, the present review clarifies an additional boundary of insight by explicitly framing VRWMP as a vehicle–road co-generated composite. Under this framing, the hazard profile of VRWMP may not be reliably inferred from tire chemistry alone, because pavement-related factors can systematically modify particle properties and exposure relevance. In particular, we emphasize that pavement materials and road construction attributes are not merely contextual modifiers; rather, they act as mechanistic determinants that influence size distributions, mineral encrustation, surface reactivity, and the bioaccessible fraction of associated constituents—thereby reshaping exposure routes and toxicity outcomes.

Building on this road engineering perspective, we identify priority knowledge gaps that are directly required for health-risk extrapolation under realistic exposure scenarios: (a) standardizable descriptors for composite particle structure (e.g., rubber core–mineral shell and associated chemical fingerprints); (b) field-relevant exposure metrics that link traffic regimes and pavement types to inhalable/ingestible fractions; and (c) model frameworks that parameterize pavement-related variables within source, transport, exposure, and effect relationships. By making these distinctions explicit, this review reduces conceptual overlap with prior VRWMP environmental-behavior summaries and proposes a more actionable research agenda that bridges road engineering, exposure science, and toxicology for VRWMP risk assessment.

## Conclusions and Outlook

### Conclusions

As a major component of non-exhaust traffic emissions, vehicle-related wear microplastics (VRWMP) represent a coupled continuum spanning particle generation, environmental transport, and ecosystem impacts. Their true environmental importance has likely been underestimated because tire-centered assessments and simulation-based emission models, often lacking sufficient in situ validation, fail to adequately capture pavement-dependent formation mechanisms and real-world exposure pathways. By emphasizing the governing role of pavement materials and surface properties, this review highlights the importance of integrating road engineering perspectives into environmental monitoring frameworks to improve ecological risk quantification and support more effective mitigation strategies.

Substantial methodological inconsistencies remain in VRWMP research, particularly in sampling approaches that differ substantially between laboratory-generated specimens from road simulators and field-based mobile collection methods. Although analytical strategies have evolved from single-component characterization toward integrated approaches employing advanced techniques such as LDIR, py-GC/MS, and TOF-SIMS, the lack of standardized testing protocols continues to hinder inter-study comparability and data reproducibility. Future methodological development should prioritize the establishment of unified sampling conditions, particle size classification criteria, pretreatment procedures, and reporting metrics. Key operational variables, including traffic load, pavement texture, tire composition, rainfall intensity, and particle aging state, should also be systematically incorporated into experimental and modeling frameworks to improve data consistency and predictive reliability.

Environmentally, VRWMP exhibits a characteristic distribution pattern in which soils function as the primary sink, while aquatic systems serve as the major transport pathway. With the increasing application of novel pavement materials, the adoption of composite tracers simultaneously reflecting contributions from both tires and pavements has become essential for accurate source apportionment. Moreover, existing transport and fate models remain inadequate in accounting for road structural heterogeneity, hydrological conditions, and climatic variability. Future studies should therefore focus on developing multi-factor coupled migration models integrating pavement characteristics, meteorological parameters, runoff dynamics, and traffic intensity. Particular attention should be directed toward the long-term transformation behavior of aged VRWMP, including fragmentation, chemical oxidation, additive leaching, and interactions with co-existing contaminants. Existing evidence demonstrates that VRWMP can induce adverse biological responses, including oxidative stress, inflammation, and DNA damage, thereby posing combined ecological and human health risks. However, current toxicological evidence remains largely based on short-term laboratory exposure scenarios, highlighting the urgent need for chronic exposure studies and environmentally realistic dose-response assessments.

Overall, this study identifies VRWMP as a critical and distinct category within the broader framework of microplastic pollution. Unlike intentionally manufactured plastics, VRWMP originates from tire wear, pavement abrasion, road markings, and braking materials, and is characterized by continuous emissions, extensive spatial distribution, and limited feasibility for end-of-pipe control. Existing evidence suggests that traffic-related wear particles may equal or even exceed consumer product-derived microplastics in several environmental compartments; however, substantial uncertainties persist regarding emission inventories, environmental fate, and risk characterization. Future research should therefore establish a more operational research agenda centered on 4 priorities: (a) standardized field-to-laboratory validation systems for emission quantification; (b) integrated physicochemical-biological assessment frameworks for evaluating aging-dependent toxicity; (c) high-resolution transport models linking road structures, drainage networks, and receiving waters; and (d) source-oriented mitigation strategies involving tire formulations, pavement design optimization, and urban runoff interception technologies. A traffic system-oriented perspective is essential for improving global microplastic inventories and informing practical interventions, including material standards for tires and pavements, resilient transport infrastructure design, and enhanced stormwater management. Given the efficient transport of VRWMP through drainage systems into downstream freshwater and marine environments, future governance strategies should also promote stronger integration across transportation, materials engineering, environmental science, and public policy sectors.

### Outlook

Building on the conclusions, future efforts must translate the identified challenges into an actionable research agenda. The following priorities, key variables, and methodological recommendations are proposed to operationalize the path forward.

1. Elucidate pavement-dependent formation mechanisms

Future work must prioritize controlled experiments to quantify how specific pavement material properties—such as asphalt binder chemistry, aggregate mineralogy, and surface texture—directly control VRWMP generation. Key investigations should systematically vary these parameters in road simulator studies, using advanced characterization techniques (e.g., SEM-EDS and py-GC/MS) to link material inputs to VRWMP outputs like EFs, size distribution, and chemical signatures. This mechanistic understanding is foundational for developing source-control strategies and predictive models.

2. Develop field-validated, intelligent emission and transport models

A critical step is building transferable models that predict real-world VRWMP fate. This requires integrating high-resolution data on traffic dynamics, pavement conditions, and meteorological variables with ground-truth VRWMP concentration data from sensor-based mobile monitoring. Methodologically, adopting a hybrid approach that combines physical transport principles with machine learning (e.g., random forest models) is recommended to create reliable, spatially resolved predictive tools for urban runoff and environmental load assessments.

3. Establish standardized protocols for sampling and analysis

To overcome the critical barrier of data comparability, the community must converge on a standardized workflow. The priority is to define and validate consensus protocols for every step—from field collection (e.g., vacuum versus sweep sampling) to laboratory analysis (e.g., density separation and instrumental identification criteria). Initiating inter-laboratory comparison studies using spiked reference materials is a concrete methodological recommendation to achieve the necessary reproducibility and harmonization across future studies.

4. Assess long-term ecological and health impacts

Research must advance beyond acute toxicity tests to evaluate the chronic, real-world risks of VRWMP. Priority questions focus on the multi-generational effects of environmentally relevant mixtures on soil biota and ecosystem functions. Methodologically, this calls for long-term mesocosm experiments with field-collected VRWMP, coupled with omics techniques (e.g., transcriptomics), to uncover sublethal biological endpoints and mechanistic pathways. This integrated approach is essential for robust ecological risk characterization.
